# Tératome mature géant rétro péritonéale

**DOI:** 10.11604/pamj.2014.18.147.4351

**Published:** 2014-06-17

**Authors:** Mouad Statoua, Abdellatif Koutani

**Affiliations:** 1 Service d'Urologie B CHU, Ibn Sina, Rabat, Maroc

**Keywords:** Tératome, péritoine, tumeur, Teratoma, peritonium, tumor

## Image en medicine

Le tératome rétro péritonéal de l'adulte est une entité très rare, sa découverte tardive s'explique par la pauvreté des signes cliniques et sa localisation rétro péritonéale profonde. Nous rapportons le cas d'une jeune patiente de 40 ans qui a consulté dans notre établissement pour des lombalgies gauches, sensation de pesanteur et une voussure abdominale, l'examen clinique retrouve une patiente en bon état générale une masse solide abdominale occupant l'hypochondre, flanc gauche et l’épigastre. Une échographie abdominale fut réalisé objectivant une masse hétérogène faisant 15 cm de grand axe, un complément scanographique met en évidence une masse rétro-péritonéale hétérogène se rehaussant partiellement après l'injection du produit de contraste et contenant des calcifications (A). devant la gène physique et esthétique rapporté par la patiente une intervention chirurgicale fut programmée et cette masse a été abordé par voie sous costale gauche, après décollement colo-pariétale gauche la masse présente des adhérences avec les structures vasculaires et en essayant d'extirper la masse une blessure de l'artère mésentérique supérieure est observée et malgré sa réparation et sa réimplantation par un greffon de la veine saphène externe, l’état de choc hémorragique n'a pas pu être contrôler malgré les transfusions massives de culots globulaire et l'introduction de drogue vasoactive, le décès de la patiente est survenu 6h après l'intervention. L'aspect macroscopique de la pièce après son exérèse met en évidence une masse solide avec une partie kystique et à son ouverture on a constaté la présence de cartilage osseux ainsi que des cheveux (B). L’étude histologique de la pièce opératoire a mis en évidence un tératome primitif mature.

**Figure 1:**
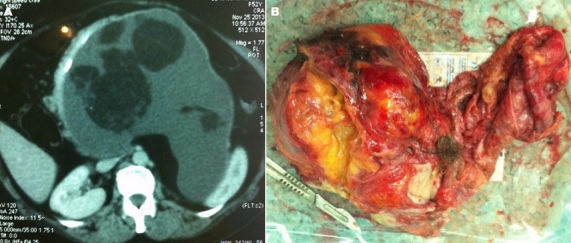
A) masse rétro péritonéale géante de 15 cm hétérogène renfermant des calcifications; B) Aspect macroscopique de la masse renfermant du tissu cartilagineux et des cheveux

